# 

**Published:** 1851-12

**Authors:** 


### WESTERN JOURNAL

OF

### MEDICINE AND SURGERY.

EDITED BY

LUNSFORD P. YANDELL, M. D.,

PROFESSOR OF PHYSIOLOGY AND PATHOLOGICAL ANATOMY IN THE UNIVERSITY OF LOUISVILLE,

AND

THEODORE S. BELL, M. D.

CXLIII.—DECEMBER, 1851.

**THIRD SERIES.**

VOL. VIII......NO. VI.

LOUISVILLE, KY:

PUBLISHED MONTHLY BY PRENTICE & WEISSINGER,

PROPRIETORS AND PRINTERS.

1851.

